# INSIGHT: Integrating Social Determinants of Health in Medical Education During Psychiatry Prison Placements

**DOI:** 10.1192/bjo.2024.321

**Published:** 2024-08-01

**Authors:** Sadia Bashir Nafees, Jakub Matusiak, Andrea Taylor-Clutton, Simon Newman, Rob Poole

**Affiliations:** 1 Bangor University, Wrexham, United Kingdom; 2 BCUHB, Wrexham, United Kingdom

## Abstract

**Aims:**

Background. The importance of the social determinants of health (SDOH) is increasingly recognised. However, medical students are taught about them as epidemiological facts. We established a programme in North Wales involving prison placements for medical students, accompanied by specific teaching to contextualise SDOH to individual patients’ mental health problems. This is being evaluated over a four-year follow-up. We report findings of qualitative evaluation of the second-year cohort.

**Methods:**

Individual interviews with students and free text data from questionnaires were analysed thematically.

**Results:**

Previous teaching about SDOH:

- “You do not understand until you see it in your own life. Lectures do not always deliver a point.”
- “Mentioned but not very explicit session like here.”

Baseline knowledge and attitudes to SDOH

- “I knew mental health and social determinants are a lot intertwined, but I would not have thought of it in such depth before coming here.”
- “I knew what SDOH were, but I have not seen it on this scale.”
- “Some students related the teaching to their personal experience of hardship*.”*

Prison placement

“I think the prison placement has given invaluable teaching about psychiatric conditions.”

“I…think it helps widen experiences in medicine, seeing a different perspective of healthcare.”

“I enjoyed the prison experience. It gave me the social aspects of health, and especially in the prison, it is clear and visible.”

Impact of the placements

“Humbling experience. A lot of patients I saw had some sort of childhood trauma.”

“Maybe I will be treating someone that is not as privileged or someone who's been in prison, so it's important…”

“Learned to have confidence when taking patient history. Do not feel awkward when asking medical questions such as suicide.”

SDOH incorporation into medical education

“Introducing the modules in medical school would be good before the students meet the patients, as the social aspect is a big part of the history.”

“These sessions need to be integrated throughout the module rather than at one point as social determinants also play a role in other specialities, not only psychiatry.”

**Conclusion:**

In previous publications, we reported positive responses to prison placements. By integrating a module about SDOH, students can develop a broader understanding of health and gain the awareness needed to address these factors in clinical practice.

